# Oncoplastic Surgery Versus Lumpectomy: Analysis of Oncological Outcomes and Surgical Complications in 1290 Breast Cancer Patients

**DOI:** 10.3390/curroncol33030163

**Published:** 2026-03-12

**Authors:** Adolfo Alejandro Lopez Rios, Michael J. Stein, Angel Arnaout, Jing Zhang

**Affiliations:** 1 Plastic Surgery Residency Program, University of Ottawa Faculty of Medicine, Box 213-1053 Carling Ave, Ottawa, ON K1Y 4E9, Canada; alopezrios@toh.ca; 2 Lenox Hill Hospital, 100 E 77th St, New York, NY 10075, USA; admin@drmichaeljstein.com; 3 Providence Healthcare, Cancer Network Interior Health, 600 West 10th Avenue, Vancouver, BC V5Z 4E6, Canada; angel.arnaout@phsa.ca; 4 Division of Plastic Surgery, The Ottawa Hospital, Ottawa, ON K1Y 4E9, Canada

**Keywords:** breast cancer, oncoplastic surgery, lumpectomy, outcomes, complications, survival

## Abstract

This 12-year retrospective study compared oncoplastic breast-conserving surgery (OBCS) with lumpectomy without reconstruction (LNR) to evaluate surgical and oncological outcomes. OBCS combines tumour removal with tissue reshaping to preserve breast contour, but concerns about treatment delays and recurrence limit its use. Among 1880 patients reviewed between 2008 and 2020, 1290 met the inclusion criteria—307 (24%) underwent OBCS and 983 (76%) underwent LNR. Women receiving OBCS were younger (mean 56 vs. 61 years, *p* < 0.0001) with similar BMIs. OBCS was associated with a slightly longer time to radiotherapy (3.93 vs. 3.57 months, *p* = 0.01) and higher rates of minor complications such as wound infection (7.17% vs. 3.66%), dehiscence (4.89% vs. 0.92%), and fat necrosis (11.73% vs. 1.12%) (all *p* < 0.0001). There were no significant differences in positive margins, mastectomy conversion, recurrence, or disease-free survival. Despite a modest delay in adjuvant therapy and increased minor complications, OBCS demonstrated equivalent oncologic safety to standard lumpectomy. These findings support OBCS as a safe breast-conserving option that maintains esthetic outcomes without compromising cancer control, encouraging its broader use in appropriately selected patients.

## Simple Summary

Oncoplastic breast-conserving surgery allows women with breast cancer to keep more of their breast while still removing the cancer safely. Although some patients worry that this approach may delay follow-up treatments or raise the risk of recurrence, this large long-term study found that while oncoplastic surgery caused slightly more minor wound issues and a small delay in starting radiotherapy, cancer control and survival outcomes were the same as with standard lumpectomy. These results suggest that the oncoplastic approach is both safe and effective, helping women achieve better cosmetic results without compromising cancer care. The findings could encourage more surgeons and patients to consider oncoplastic surgery as a standard option, guide health systems in planning breast cancer services, and support future research on optimizing outcomes and recovery after breast-conserving surgery.

## 1. Introduction

Oncoplastic breast-conserving surgery (OBCS) has similar oncological outcomes [1,2,3,4,5] but improved health-related quality of life [6,7,8], psychosocial satisfaction [9,10,11], and esthetic results compared to lumpectomy alone [12,13,14]. The indications for OBCS are expanding [15,16,17,18] and now include patients who historically underwent mastectomy, such as women with large tumours (>4 cm) [19], locally advanced cancers [20,21], those who underwent prior neoadjuvant chemotherapy for more invasive tumours [22], and patients with synchronous multicentric and multifocal breast cancers [23].

The potential for delays in adjuvant therapy may influence decision-making when women are offered lumpectomy alone versus lumpectomy and oncoplastic rearrangement. However, the selection of surgical technique is more frequently guided by tumour characteristics, including size and location, as well as the ability to achieve adequate oncologic resection while preserving breast shape and achieving satisfactory esthetic outcomes [24,25]. OBCS relies on techniques adopted from reduction mammoplasty and locoregional flaps; thus, patients are subject to an extended surgical intervention, which could increase the risk of surgical complications such as seroma, hematoma, and infection [26,27]. OBCS procedures were classified according to established oncoplastic principles based on the volume of tissue resected and the complexity of reconstruction. Level I techniques involved limited glandular mobilization and local tissue rearrangement following small-volume resections (<20% of breast volume), including rotational flaps, advancement flaps, and mastopexy-type closures. Level II techniques involved larger resections (approximately 20–50% of breast volume) requiring more extensive breast reshaping through volume-displacement techniques such as therapeutic mammoplasty or other local flap-based reconstructions. Wound healing complications can delay the onset of postoperative chemotherapy and radiation and, in doing so, theoretically impact survival rates [28,29].

While some studies have demonstrated a higher minor complication rate among patients receiving OBCS compared to those undergoing lumpectomy [30,31], others have reported similar complication rates [32,33]. Additionally, studies have found comparable timing to adjuvant therapy, as well as similar rates of positive margins, re-excisions, and disease-free survival [34,35].

The objective of the present review was to retrospectively review surgical and oncologic outcomes of lumpectomy cases, with and without oncoplastic rearrangement.

## 2. Materials and Methods

### 2.1. Study Design and Setting

Research Ethics Board (REB) approval (Protocol: 20200388-01H) was obtained for a retrospective chart review study. Data were collected from a prospectively maintained database of breast cancer patients at The Ottawa Hospital. All patients who received oncoplastic surgery with or without contralateral balancing reduction (OBCS) or lumpectomy with no local reconstruction (LNR) between 1 January 2008 and 1 June 2020 were considered for inclusion. This study adhered to the STROBE guideline for reporting observational studies.

### 2.2. Study Participants and Eligibility Criteria

All women over the age of 18 with breast cancer treated with OBCS and LNR at The Ottawa Hospital were retrospectively reviewed for eligibility. Consecutive patients receiving either OBCS with or without contralateral balancing reduction or mastopexy or curative-intent lumpectomy with no local reconstruction were included [36]. All patients undergoing lumpectomy, mastectomy, or therapeutic mammoplasty for risk-reduction alone or in the form of lone surgical biopsies were excluded. Patients with distant metastatic disease, stage IV cancer, or delayed breast reconstruction were also excluded.

### 2.3. Data Collection

Data collection was performed independently by two reviewers, and a third reviewer resolved any discrepancies in outcome reporting. Patient baseline characteristics included age, body mass index (BMI), ASA status (Grade I-IV), smoking status (current, former, or non-smoker), and comorbidities (hypertension, ischemic heart disease, diabetes mellitus, COPD, asthma, stroke, arthritis, and dementia). Treatment characteristics included the level of OBCS (I–II) technique (rotational, advancement flap closures, mastopexy, mammoplasty, local flap), incision pattern (wise, vertical, peri areolar), pedicled planning of NAC (superior, inferior, central, superomedial, superolateral pedicles), and presence of contralateral balancing reduction. All our patients received prophylactic antibiotics with cefazolin, repeated every 4 h intraoperatively if the surgery was prolonged. In cases of serious β-lactam allergy, we use clindamycin or vancomycin before incision [9,37,38,39]. Tumour characteristics included size, weight of specimen, location (left, right, bilateral), laterality, focality, and node dissection type (sentinel, axillary, axillary sentinel, radical axillary). Clinicopathologic data included invasive disease grade (1, 2, 3), histologic grade (1, 2, 3), tumour stage (T1a, T1b, T1c, T2, T3), nodal status (N0, N1, not reported), and malignancy status.

### 2.4. Outcomes of Interest

Primary outcomes included timing to adjuvant radiotherapy and chemotherapy in months. Secondary outcomes included positive margins, mastectomy conversion, locoregional recurrence, metastasis, revision surgery, major (re-trip to OR) complications, minor complications (seroma, hematoma, infection, wound dehiscence, NAC necrosis, fat necrosis clinically), and disease-free survival (DFS). Major complications were defined as any complication requiring readmission after hospital discharge or unplanned reoperation within 6 weeks of OBCS or LNR or prior to the start of adjuvant radiotherapy or chemotherapy [40]. Minor complications were defined as those managed conservatively. Time to locoregional recurrence was defined as the time interval between the date of breast surgery and the date of first documented locoregional recurrence. DFS was calculated as the time from the date of breast cancer surgery to the date of locoregional recurrence. Follow-up time for all patients was recorded. There were no patients lost to follow-up, and the study’s last follow-up date was set as 31 December 2021.

### 2.5. Statistical Analysis

Continuous variables were compared using an independent *t*-test. Categorical variables were compared between treatment groups using either the Chi-square test or Fisher’s exact test, where appropriate. Kaplan–Meier plots were created, and a log-rank test was applied to compare DFS between groups. The time from breast cancer surgery to disease recurrence provided an estimate of DFS probability. An independent *t*-test was performed to compare means, and a Wilcoxon rank-sum test was performed to compare medians between groups. For all statistical tests, a two-tailed test was used to determine significance at the 5% level. A value of *p* < 0.05 was considered statistically significant in all cases. We reported means with standard deviations or medians with interquartile ranges for continuous variables, and numbers with percentages for categorical variables. All statistical analyses were performed using SAS version 9.4 for Windows (SAS Institute Inc., Cary, NC, USA). Variables not reported in the medical records were considered missing and appropriately accounted for in the analysis.

## 3. Results

**Figure 1:**
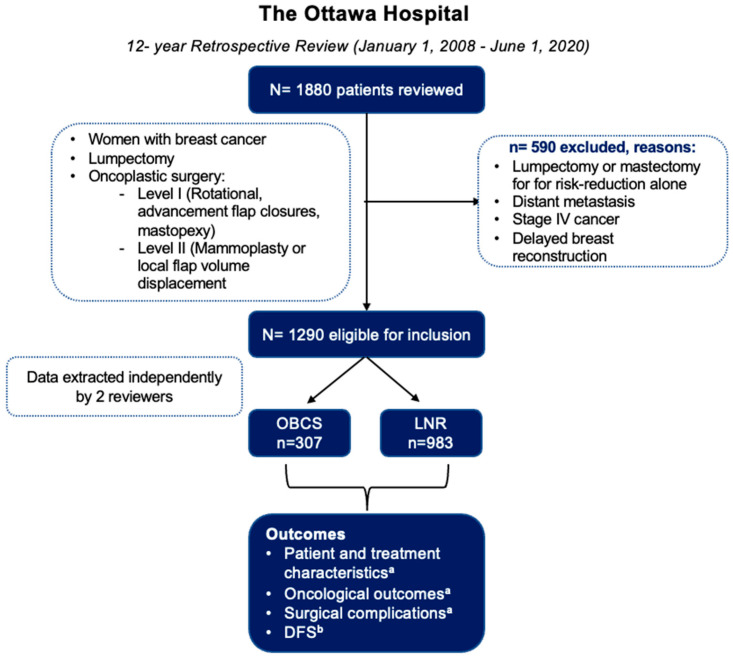
Patient flow diagram (OBCS, oncoplastic breast-conserving surgery; LNR, lumpectomy with no reconstruction). ^a^ Independent *t*-test for difference in means (SD) or Chi-square (χ^2^) test for difference in proportions. ^b^ Survival analysis using Kaplan–Meier methods.

Of 1880 breast cancer patients screened, 1290 (69%) patients met the inclusion criteria, and 590 (31%) patients were excluded due to metastatic disease, stage IV cancer, recurrent breast cancer, or delayed breast reconstruction (Figure 1). The cohort consisted of 307 (24%) patients who underwent OBCS and 983 (76%) patients who underwent LNR. In the OBCS group, 150 (49%) patients received Level 1 OBCS (rotational flap, advancement flap closures, mastopexy procedures),157 (51%) received Level 2 OBCS (mammoplasty, local flap volume displacement procedures), and 152 (49.5%) received contralateral balancing symmetrisation (Table 1).

### 3.1. Patient Demographics

Patient demographics are summarized in Table 2. Compared to the LNR group, patients in the OBCS group were younger [OBCS: 56.5 years (±11.7) vs. LNR: 61.1 years (±12.0), *p* < 0.001] and had a similar BMI [OBCS: 28.6 (±6.3) vs. LNR: 27.9 (±6.3), *p* > 0.05]. OBCS patients had lower rates of hypertension [OBCS: 77 (25.1%) vs. LNR: 328 (33.4%), *p* < 0.0001], lower rates of cardiovascular disease [OBCS: 32 (10.4%) vs. LNR: 182 (18.5%), *p* < 0.001], and lower rates of diabetes mellitus [OBCS: 17 (5.5%) vs. LNR: 95 (9.7%), *p* < 0.001]. There were no significant differences in the rates of non-smokers, former, or current smokers, COPD, dementia, asthma, arthritis, stroke, or ASA status between OBCS and LNR (*p* > 0.0.5)

Tumour characteristics are summarized in Table 3. Both the size [OBCS: 2.58 (2.45) cm vs. LNR: 1.86 (1.11) cm] and weight [OBCS: 427 (276.9) grams vs. LNR: 50.5 (19.1) grams] of tumours recovered from patients who underwent OBCS were significantly greater than tumours in the LNR group (*p* < 0.0001). There was a higher proportion of patients in the OBCS group who had multifocal breast lesions compared to the LNR [OBCS: 77 (27.6%) vs. LNR: 93 (11.71%), *p* < 0.001]. The distribution of tumour stage across OBCS and LNR groups was significantly different (*p*< 0.001) as patients in OBCS had more T2 [131 (42.6%)] and T3 [19 (6.19%)] tumours compared to LNR [T2: 284 (28.9%) and T3: 13 (1.32%)]. Patients who underwent LNR had more T1a-c tumours [T1a: 62 (6.31%), T1b:114 (11.6%), T1c: 351 (35.7%)] compared to patients who received OBCS [T1a: 15 (4.89%), T1b: 26 (8.47), T1c: 83 (27%). There were more OBCS patients who had N1 [75 (24.4%)] and N2 [14 (4.56%)] nodal status compared to LNR [N1: 165 (16.8%) and N2: 26 (2.64%), *p* = 0.0088].

### 3.2. Surgical Complications

Ten (0.78%) patients experienced a major complication, with no difference between groups [OBCS: 4 (1.3%) vs. LNR: 6 (0.61%), *p* > 0.05] (Table 4). The OBCS group had a significantly greater proportion of patients who experienced a minor complication compared to the LNR group [OBCS: 130 (42.3%) vs. LNR: 227 (23.1%), *p* < 0.001]. OBCS patients had higher rates of wound infection [OBCS: 22 (7.17%) vs. LNR 36 (3.66%), *p* = 0.0097], dehiscence [OBCS: 15 (4.89%) vs. 9 (0.92%), *p* < 0.001], and fat necrosis [OBCS: 36 (11.73%) vs. LNR: 11 (1.12%), *p* < 0.001]. In the OBCS group, more minor complications occurred in Level II OBCS vs. Level I [Level I: 45 (14.7%) vs. Level II: 86 (28.0%)]. There were no statistically significant differences in the rates of seroma, hematoma, or NAC necrosis across treatment groups (*p* > 0.05)

### 3.3. Timing of Adjuvant Therapy

A total of 123 (40.0%) OBCS and 309 (31.43%) LNR patients received adjuvant chemotherapy and 283 (92.18%) OBCS and 871 (88.60%) LNR patients received adjuvant radiotherapy (Table 5). Compared to LNR, OBCS was associated with a significantly longer time to adjuvant radiotherapy [OBCS: 3.93 (±2.28) months vs. LNR: 3.57 (±2.14) months, *p* = 0.0153]. There were no statistically significant differences in the timing to adjuvant chemotherapy between OBCS and LNR patients [OBCS: 1.85 (±0.72) vs. LNR: 1.91 (±0.79), *p* > 0.05]. According to our institutional breast cancer treatment protocols, patients needing adjuvant chemotherapy usually received chemotherapy before adjuvant radiotherapy, while those not requiring chemotherapy went directly to radiotherapy. In our cohort, 40.1% of patients in the OBCS group and 31.4% in the LNR group received adjuvant chemotherapy, with the rest proceeding straight to radiotherapy. Although a slightly higher percentage of patients in the OBCS cohort received adjuvant chemotherapy, the time to initiation was similar across groups, indicating that treatment sequencing did not differ markedly in clinical practice.

### 3.4. Oncological Outcomes

Overall, 1220 (94.6%) patients were alive at the last follow-up [OBCS: 288 (93.8%) vs. LNR: 932 (94.8%), *p* = 0.4993]. The median follow-up for the entire cohort was 5.32 years (SD 2.78), with a median of 5.54 years (SD 2.90) for the lumpectomy-alone (LNR) group and 4.64 years (SD 2.22) for the oncoplastic breast-conserving surgery (OBCS) group (*p* < 0.0001). Among the OBCS subgroups, median follow-up was 5.36 years (SD 2.25) for Level I procedures and 3.96 years (SD 1.97) for Level II procedures. There were 11 (3.58%) locoregional recurrences in OBCS and 40 (4.07%) in LNR, with no significant differences between the groups. The rates of positive margins [OBCS: 32 (10.42%) vs. LNR: 99 (10.07%), *p* = 0.8435] and distant metastasis [OBCS: 19 (6.19%) vs. LNR: 45 (4.58%), *p* = 0.2564] were demonstrably similar between OBCS and LNR. In the OBCS group, two patients (0.65%) underwent two revision surgeries, and 25 patients (8.14%) underwent one revision surgery. In the LNR group, four patients (0.41%) underwent two revision surgeries, and 69 patients (7.02%) underwent one revision surgery. These revision surgeries were performed for esthetic or reconstructive purposes, such as improving symmetry or contour, and do not include re-excisions for oncological reasons. Overall, there was no significant difference in the revision surgery rate between the OBCS and LNR groups (*p* = 0.6131).

### 3.5. Disease-Free Survival

**Figure 2:**
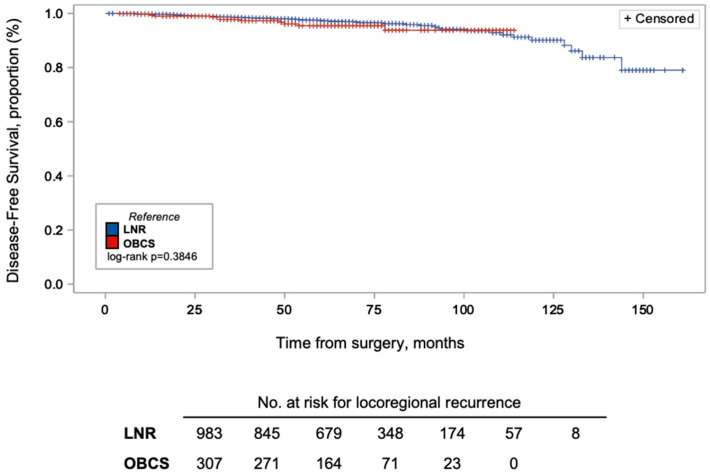
Kaplan–Meier curves of disease-free survival (DFS) proportion (%) by breast surgery group. DFS were similar between OBCS and LNR (log-rank *p* = 0.3846). Vertical lines on the DFS curve indicate censored patients are shown using vertical marks (96% of patients were censored at the time of data cut-off).

**Figure 3:**
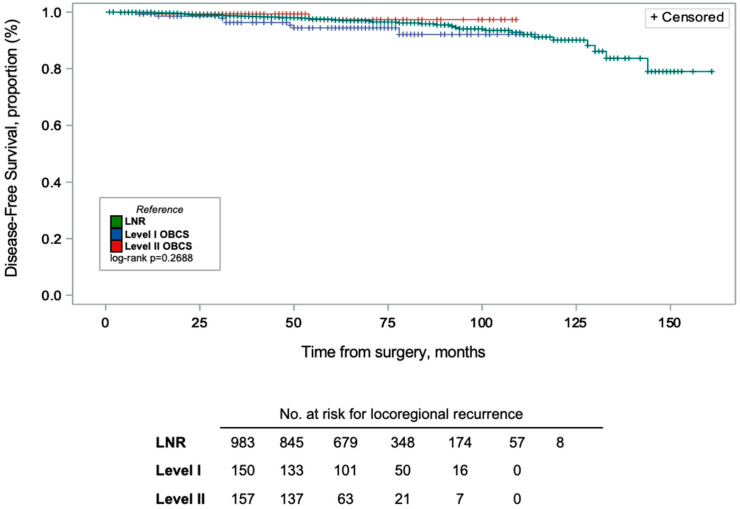
Kaplan–Meier curves of disease-free survival (DFS) proportion (%) by breast surgery group. DFS was similar between both OBCS types and LNR (log-rank *p* = 0.2688). Vertical lines on the DFS curve indicate censored patients (96% of patients were censored at the time of data cut-off).

Within the OBCS group, DFS was also compared between Level I and Level II OBCS procedures (Figure 2 and Figure 3). Kaplan–Meier analysis showed that, compared to LNR, OBCS had no statistically significant difference in DFS (log-rank *p* = 0.3846), as well as no significant differences between Levels I and II OBCS (log-rank *p* = 0.2688). Overall, there were a total of 50 recurrences across groups, and 96% of patients were censored at the time of the data cut-off (31 December 2021).

## 4. Discussion

The indications for OBCS continue to expand, with increasing evidence supporting its surgical and oncologic safety. Nevertheless, many patients still elect to undergo lumpectomy alone or completion mastectomy due to the fear of recurrence and delay in cancer treatment that can impact survival [18,41].

Previous studies have investigated whether surgical and oncologic outcomes differ between these procedures. Several large-scale studies have variably shown both lower [32,42,43] and similar complication rates in OBCS compared to LNR [5,14,33,44,45,46,47]. In contrast, some studies have demonstrated higher rates of minor and major complications [48,49]. Our study assessed 1290 breast cancer patients, of whom 10 experienced a major complication that necessitated a re-visitation to the OR due to seroma or hematomas requiring aspiration, percutaneous drain placement, or operative debridement. Differences in the rates of major complications between OBCS and LNR were non-significant and similar overall. However, rates of minor complications, including wound dehiscence, wound infection, and fat necrosis, were found to be higher in OBCS. As such, our study demonstrates that OBCS can be associated with higher minor complication rates. While the reason for such observation is unclear, patients who undergo OBCS are subjected to greater degrees of tissue rearrangement and longer operative times. Therefore, depending on the specific technique of OBCS utilized, procedures can be complex, lengthy, and associated with higher postoperative complication rates [37,50,51]. These findings align with our study’s result, which also demonstrates higher relative minor complication rates in Level II versus Level I OBCS procedures. An additional factor that may influence postoperative complications is the use of neoadjuvant therapy. In our cohort, patients undergoing OBCS were more likely to receive neoadjuvant systemic therapy compared to those treated with LNR. Although the existing literature presents conflicting evidence regarding the impact of neoadjuvant treatment on wound healing and postoperative complications, this discrepancy may introduce a potential confounding factor that contributes to the higher rate of minor complications observed in the OBCS group. Similarly, patients who underwent OBCS were also offered symmetry procedures such as mammaplasty and mastopexy on the contralateral side, of which nearly half (49.5%) pursued this option. Nearly half of patients undergoing OBCS elected to undergo a contralateral symmetry procedure. However, complications in our dataset were recorded at the patient level and were not attributed to a specific breast or surgical component. Therefore, we were unable to determine whether complications originated from the oncologic procedure or the contralateral symmetry surgery. Future prospective studies with procedure-specific complication tracking would help clarify the contribution of symmetry procedures to postoperative morbidity.

Regarding the timing of adjuvant care, concerns about possible delays to adjuvant chemotherapy and radiotherapy have been widely discussed. Numerous studies have sought to investigate the impact of OBCS on timing to adjuvant care. Studies have shown that when complications do occur in the oncoplastic group, the time to initiation of radiation therapy is often extended [18,52]. However, several studies have shown no difference in timing or delay to either adjuvant chemotherapy or radiotherapy regardless of the presence of complications [14,17,46,53,54]. Our study demonstrated slightly longer mean times to adjuvant radiotherapy (3.93 vs. 3.57 months) in the OBCS group, but no significant differences in timing to adjuvant chemotherapy. Although a higher proportion of patients in the OBCS cohort received adjuvant chemotherapy, the sequencing of adjuvant therapy followed standard institutional protocols, with chemotherapy administered prior to radiotherapy when indicated. While very few studies specifically comment on the relationship between complications and the timing of chemotherapy or radiation, any potential delay in adjuvant radiotherapy for patients in our study can largely be explained by minor complications that required appropriate treatment prior to the safe commencement of radiotherapy.

With respect to long-term oncological outcomes, oncological safety data supporting oncoplastic rearrangement in standard breast cancer management are well-established. To date, numerous studies have demonstrated no difference in recurrence rates [55,56,57], positive margins [58,59], metastasis [60,61], or disease-free survival [19,52] between OBCS and LNR. Rates of revision surgery [60] and completion mastectomies [61,62] have also been reported to be significantly lower in individuals who receive OBCS. Our study demonstrated no differences in rates of locoregional recurrence, positive margins, distant metastasis, or revision surgery. Similarly, with approximately 10 years of follow-up data, our study also showed no significant differences in DFS between OBCS and LNR. Therefore, the collective findings of our study provide further support for the oncological safety of OBCS as a treatment option for appropriately selected breast cancer patients.

This present study is limited by the differences between treatment groups, which were partly unavoidable given that specific patient characteristics, breast morphologies, and tumour characteristics made some women more likely to pursue an oncoplastic approach. The retrospective nature of our study also limited our ability to control for biases that could have impacted the results, including the potential impact of neoadjuvant therapy, as a significant proportion of patients in the OBCS group received this treatment during their care.

Overall, oncoplastic surgery aims to bridge the gap between lumpectomy and mastectomy with or without reconstruction by maximizing tumour margins while maintaining breast form and function. To our knowledge, this is the largest-scale Canadian analysis of patients undergoing oncoplastic reconstruction. Recognition and understanding of current evidence surrounding possible delays to adjuvant therapy and their long-term implications on oncological safety are necessary. Factors such as longer operative time, increased risk of complications with pertinent sequelae affecting quality of life, and potential delay in adjuvant therapy should all be carefully considered in a patient’s surgical decision-making. Surgeons should also continue to practice shared decision-making with their patients by outlining the pros and cons of each procedure and guiding patients to make an informed decision based on their own comorbidities, the size and location of their tumours, the extent of resection, and their comfort level [63].

## 5. Conclusions

Women who underwent OBCS compared to LNR had higher postoperative minor complication rates and slightly longer times to first adjuvant radiation therapy. However, OBCS is not associated with an increased risk of locoregional recurrence or inferior long-term disease-free survival. This large-scale study further supports the oncological safety of OBCS and can be used to help educate patients as they compare treatment options.

## Figures and Tables

**Table 1:** Treatment characteristics (OBCS vs. LNR).

	No. Patients (%)
LNR	983 (76.2)
OBCS	307 (23.8)
Level I	150 (48.9)
Rotational flap closure	31 (10.1)
Advancement flap closure	107 (34.9)
Round block/doughnut mastopexy	11 (3.58)
Crescent mastopexy	2 (0.65)
Racket method mastopexy	3 (0.98)
Level II	157 (51.1)
Mammoplasty	153 (49.8)
Local flap volume displacement	28 (9.12)
Contralateral Balancing	152 (49.5)

**Acronyms**: OBCS, oncoplastic breast-conserving surgery; LNR, lumpectomy with no reconstruction.

**Table 2:** Patient demographics (OBCS vs. LNR).

	No. Patients *n* = 1290	LNR *n* = 983	OBCS *n* = 307	*p*-Value
^a^ Age, years (SD)	60 (12.07)	61.11 (11.98)	56.45 (11.69)	<0.0001 *
^a^ BMI, kg/m^2^ (SD)	28.12 (6.36)	27.95 (6.36)	28.62 (6.33)	0.1126
^b^ Smoking status, no. (%)				
Non-smoker	803 (62.2)	608 (62.4)	195 (64.1)	
Former smoker	373 (28.9)	289 (29.7)	84 (27.6)	0.7910
Current smoker	102 (7.90)	77 (7.91)	25 (8.22)	
Not reported	12 (0.93)	9 (0.92)	3 (0.98)	
^b^ Comorbidities, no. (%)				
Hypertension	405 (31.4)	328 (33.4)	77 (25.1)	0.0006 *
Cardiovascular disease	214 (16.6)	182 (18.5)	32 (10.4)	<0.0001 *
Stroke	6 (0.47)	5 (0.51)	1 (0.33)	1.0
Diabetes mellitus	112 (8.68)	95 (9.67)	17 (5.54)	0.0247 *
COPD	62 (4.81)	46 (4.68)	16 (5.21)	0.7062
Asthma	154 (11.9)	115 (11.7)	39 (12.7)	0.6397
Arthritis	392 (30.4)	310 (31.5)	82 (26.7)	0.1063
Dementia	4 (0.31)	4 (0.41)	0 (0.0)	0.5781
^b^ ASA Status, no. (%)				
I	83 (6.43)	61 (6.21)	22 (2.24)	
II	731 (56.7)	540 (54.9)	191 (62.2)	0.4612
III	372 (28.8)	281 (28.6)	91 (29.6)	
IV	13 (1.0)	12 (1.22)	1 (0.33)	
Not reported	91 (7.05)	89 (9.05)	2 (0.65)	
^a^ Follow-up, years (SD)	5.32 (2.78)	5.54 (2.90)	4.64 (2.22)	<0.0001 *
Level I			5.36 (2.25)	<0.0001 *
Level II			3.96 (1.97)	

Acronyms: BMI, body mass index; OBCS, oncoplastic breast-conserving surgery; LNR, lumpectomy with no reconstruction; No, number; SD, standard deviation. ^a^ Independent *t*-test for the difference in means (SD). ^b^ Chi-square used to compare differences in proportions (or Fisher’s exact for counts < 5). * Indicates statistical significance at *p* < 0.05. Comparisons in the Level of OBCS were made between the two subgroups.

**Table 3:** Tumour and clinicopathological characteristics (OBCS vs. LNR).

	No. Patients *n* = 1290	LNR *n* = 983	OBCS *n* = 307	*p*-Value
^a^ Size, cm (SD)	2.04 (1.58)	1.86 (1.11)	2.58 (2.45)	<0.0001 *
^a^ Weight, g (SD)	418.3 (276.9)	50.5 (19.1)	427.0 (276.9)	<0.0001 *
^b^ Location, no. (%)				
Left	656 (50.9)	498 (50.7)	158 (51.5)	
Right	618 (47.9)	475 (48.3)	143 (46.6)	0.4032
Bilateral	16 (1.24)	10 (1.02)	6 (1.95)	
^b^ Breast cancer laterality, no. (%)				
Unilateral lesion	1151 (89.2)	967 (98.4)	184 (59.9)	<0.0001 *
Bilateral lesions	139 (10.8)	16 (1.63)	123 (40.1)	
^b^ Breast lesion focality, no. (%)				
Unifocal	903 (70.0)	701 (88.3)	202 (72.4)	
Multifocal	170 (13.2)	93 (11.7)	77 (27.6)	<0.0001 *
Not reported	217 (16.8)	74 (7.53)	26 (8.47)	
^b^ Lymph node procedure type, no. (%)				
None	193 (14.9)	168 (17.1)	25 (8.14)	
Sentinel lymph node biopsy	1016 (78.8)	765 (77.8)	251 (81.8)	0.0164 *
Axillary dissection	81 (6.28)	50 (5.09)	31 (10.1)	
^b^ Invasive disease grade, no. (%)				
Grade 1	235 (18.2)	187 (20.4)	48 (16.1)	
Grade 2	495 (38.4)	388 (42.4)	107 (35.9)	0.0041 *
Grade 3	484 (37.5)	341 (37.2)	143 (47.9)	
Not reported	76 (5.89)	67 (6.82)	9 (2.93)	
^b^ Histological type, no. (%)				
DCIS	194 (15.0)	168 (17.1)	26 (8.46)	
LCIS	99 (7.67)	71 (7.22)	28 (2.85)	
IDC	752 (58.3)	578 (58.8)	204 (66.4)	0.5755
ILC	133 (10.3)	92 (9.36)	41 (13.4)	
Mixed	112 (8.68)	74 (7.53)	38 (12.4)	
^b^ Receptor status, no. (%)				
ER +	983 (76.2)	758 (77.1)	225 (73.3)	0.3750
PR +	875 (67.8)	680 (69.2)	195 (63.5)	0.1430
HER-2 +	124 (9.61)	89 (9.05)	35 (11.4)	0.3113
^b^ Tumour stage, no. (%)				
Tis	103 (7.98)	84 (8.54)	19 (6.19)	
T1a	77 (5.97)	62 (6.31)	15 (4.89)	
T1b	140 (10.85)	114 (11.6)	26 (8.47)	
T1c	434 (33.6)	351 (35.7)	83 (27.0)	<0.0001 *
T2	415 (32.2)	284 (28.9)	131 (42.6)	
T3	32 (2.48)	13 (1.32)	19 (6.19)	
T4	2 (1.55)	0 (0)	2 (0.65)	
Not reported	87 (6.74)	75 (7.63)	12 (3.91)	
^b^ Tumour node, no. (%)				
NX	108 (8.37)	92 (9.36)	16 (5.21)	
N0	765 (59.3)	579 (58.9)	186 (60.6)	
N1	240 (18.6)	165 (16.8)	75 (24.4)	0.0088 *
N2	40 (3.10)	26 (2.64)	14 (4.56)	
N3	14 (1.09)	8 (0.81)	6 (1.95)	
Not reported	123 (9.53)	113 (11.5)	10 (3.26)	

Acronyms: OBCS, oncoplastic breast-conserving surgery; LNR, lumpectomy with no reconstruction; ER, estrogen receptor; HER-2, human epidermal growth factor receptor; PR, progesterone receptor; No, number; SD, standard deviation. ^a^ Independent *t*-test for the difference in means (SD). ^b^ Chi-square used to compare differences in proportions (or Fisher’s exact for counts < 5). * Indicates statistical significance at *p* < 0.05.

**Table 4:** Post-surgical complications (OBCS vs. LNR).

	No. Patients *n* = 1290	LNR *n* = 983	OBCS *n* = 307	*p*-Value
^b^ Major complications (requiring surgery or readmission), no (%)	10 (0.78)	6 (0.61)	4 (1.30)	0.2613
Level I			0 (0.0)	
Level II			4 (1.30)	
^b^ Seroma, no. (%)	179 (13.9)	139 (14.1)	40 (13.0)	0.6230
Level I			25 (8.14)	
Level II			15 (4.89)	
^b^ Hematoma, no. (%)	48 (3.72)	33 (3.36)	13 (4.23)	0.2166
Level I			8 (2.61)	
Level II			7 (2.28)	
^b^ Infection, no. (%)	58 (4.50)	36 (3.66)	22 (7.17)	0.0097 *
Level I			5 (1.63)	
Level II			18 (5.86)	
^b^ Wound dehiscence, no. (%)	24 (1.86)	9 (9.18)	15 (4.89)	<0.0001 *
Level I			1 (0.33)	
Level II			14 (4.56)	
^b^ Fat necrosis, no. (%)	47 (3.64)	36 (3.66)	11 (3.58)	<0.0001 *
Level I			6 (1.95)	
Level II			30 (9.77)	
^b^ NAC necrosis, no. (%)	2 (0.16)	0 (0.0)	2 (0.65)	0.0565
Level I			0 (0.0)	
Level II			2 (0.65)	

Acronyms: OBCS, oncoplastic breast-conserving surgery; LNR, lumpectomy with no reconstruction; NAC, nipple–areola complex; No, number. ^b^ Chi-square used to compare differences in proportions (or Fisher’s exact for counts < 5). * Indicates statistical significance at *p* < 0.05.

**Table 5:** Neoadjuvant and adjuvant therapy timing (OBCS vs. LNR).

	No. Patients *n* = 1290	LNR *n* = 983	OBCS *n* = 307	*p*-Value
^b^ Neoadjuvant chemotherapy, no. (%)	59 (4.57)	29 (2.95)	30 (9.77)	<0.0001 *
^a^ Time from start of therapy to surgery, months (SD)	4.71 (1.1)	4.76 (1.21)	4.67 (0.99)	0.7511
Level I			4.33 (0.82)	0.5677
Level II			4.75 (1.03)	
^b^ Adjuvant chemotherapy, no. (%)	432 (33.5)	309 (31.4)	123 (40.1)	0.0052 *
^a^ Time from surgery to start of therapy, months (SD)	1.9 (0.77)	1.91 (0.79)	1.85 (0.72)	0.4722
Level I			180 (0.76)	0.4719
Level II			1.89 (0.69)	
^b^ Adjuvant radiotherapy, no. (%)	1154 (89.5)	871 (88.6)	283 (92.2)	0.0749
^a^ Time from surgery to start of therapy, months (SD)	3.66 (2.18)	3.57 (2.14)	3.93 (2.28)	0.0153 *
Level I			3.68 (2.21)	0.0758
Level II			4.16 (2.32)	

Acronyms: OBCS, oncoplastic breast-conserving surgery; LNR, lumpectomy with no reconstruction; No, Number; SD, Standard deviation. ^a^ Independent *t*-test for the difference in means (SD). ^b^ Chi-square used to compare differences in proportions (or Fisher’s exact for counts < 5). * Indicates statistical significance at *p* < 0.05. Comparisons in Level of OBCS were made between the two subgroups.

## Data Availability

The data presented in this study are available on request from the corresponding author due to privacy, legal, and ethical restrictions.

## Abbreviations

The following abbreviations are used in this manuscript:

BMI	Body Mass Index
COPD	Chronic Obstructive Pulmonary Disease
DFS	Disease-Free Survival
ER	Estrogen Receptor
Her-2	Human Epidermal Growth Factor Receptor
LNR	Lumpectomy Without Reconstruction
NAC	Nipple–Areola Complex
No	Number
OBCS	Oncoplastic Breast-Conserving Surgery
PR	Progesterone Receptor
SD	Standard Deviation
